# A Novel Fully Automated Molecular Diagnostic System (AMDS) for Colorectal Cancer Mutation Detection

**DOI:** 10.1371/journal.pone.0062989

**Published:** 2013-05-09

**Authors:** Shiro Kitano, Jamie Myers, Junko Nakamura, Akio Yamane, Mami Yamashita, Masato Nakayama, Yusuke Tsukahara, Hiroshi Ushida, Wanqing Liu, Mark J. Ratain, Masahiko Amano

**Affiliations:** 1 Technical Research Institute, Toppan Printing Co., Ltd., Chiba, Japan; 2 Section of Hematology/Oncology, Department of Medicine, The University of Chicago, Chicago, Illinois, United States of America; 3 RIKEN GENESIS, Kanagawa, Japan; The Chinese University of Hong Kong, Hong Kong

## Abstract

**Background:**

*KRAS, BRAF* and *PIK3CA* mutations are frequently observed in colorectal cancer (CRC). In particular, *KRAS* mutations are strong predictors for clinical outcomes of EGFR-targeted treatments such as cetuximab and panitumumab in metastatic colorectal cancer (mCRC). For mutation analysis, the current methods are time-consuming, and not readily available to all oncologists and pathologists. We have developed a novel, simple, sensitive and fully automated molecular diagnostic system (AMDS) for point of care testing (POCT). Here we report the results of a comparison study between AMDS and direct sequencing (DS) in the detection of *KRAS, BRAF* and *PI3KCA* somatic mutations.

**Methodology/Principal Finding:**

DNA was extracted from a slice of either frozen (n = 89) or formalin-fixed and paraffin-embedded (FFPE) CRC tissue (n = 70), and then used for mutation analysis by AMDS and DS. All mutations (n = 41 among frozen and 27 among FFPE samples) detected by DS were also successfully (100%) detected by the AMDS. However, 8 frozen and 6 FFPE samples detected as wild-type in the DS analysis were shown as mutants in the AMDS analysis. By cloning-sequencing assays, these discordant samples were confirmed as true mutants. One sample had simultaneous “hot spot” mutations of *KRAS* and *PIK3CA*, and cloning assay comfirmed that E542K and E545K were not on the same allele. Genotyping call rates for DS were 100.0% (89/89) and 74.3% (52/70) in frozen and FFPE samples, respectively, for the first attempt; whereas that of AMDS was 100.0% for both sample sets. For automated DNA extraction and mutation detection by AMDS, frozen tissues (n = 41) were successfully detected all mutations within 70 minutes.

**Conclusions/Significance:**

AMDS has superior sensitivity and accuracy over DS, and is much easier to execute than conventional labor intensive manual mutation analysis. AMDS has great potential for POCT equipment for mutation analysis.

## Introduction

The human *KRAS* oncogene is mutated in over 30% of CRC, and more than 3,000 point mutations have been reported to date [1]. The most frequent alterations are detected in codon 12 (∼82% of all reported *KRAS* mutations) and in codon 13 (∼17%), which are both in exon 2 of the *KRAS* gene [2] and appear to play a major role in the progression of CRC [3]. *BRAF* encodes a serine/thereonine kinase that activates the RAS-MAPK pathway, and its mutation have been found in 4–15% of CRC. *PIK3CA* encodes the catalytic subunit p110 alpha of PI3K [4], and mutated PIK3CA stimulates the AKT pathway and promotes cell growth in various cancers, including CRC [5]. *PIK3CA* mutations have been described in 10%–30% of CRC [6], and are associated with *KRAS* mutation. There has been a report that the presence of mutations in *PIK3CA*, *KRAS*, or *BRAF* in CRC showed worse patient outcome [7], and among patients who undergo a curative resection of CRC, *PIK3CA* mutation is associated with shorter cancer-specific survival [8]. However, the adverse effect of *PIK3CA* mutation may be potentially limited to patients with *KRAS* wild-type tumors [8].

Cetuximab and panitumumab are effective epidermal growth factor receptor (EGFR) targeted agents for metastatic colorectal cancer (mCRC), but patients whose tumors have *KRAS* mutations except G13D [9] are generally believed to not benefit from these agents [10], [11]. Furthermore, mutations in *BRAF* and *PIK3CA* have also been reported to affect the efficacy of EGFR-targeted agents [12], [13]. Given the important value of these mutations in prediction of clinical outcome in mCRC patients, a rapid, reliable and sensitive technique simultaneously detecting them would be essential for informed pharmacotherapy. Thus far, although many technologies have been developed, they are limited by the complicated procedure, high cost, low throughput or other issues. For example, direct Sanger sequencing (DS) is currently still considered as a gold standard for detecting these mutations. However, the DS method requires multiple steps, lacking a capability for automated analysis. It also has a long turn-around-time and is overall relatively expensive compared to other methods. Other newly developed methods including PCR-related technologies [14]–[17], sequencing platforms [18], [19], and other methods such as HRM (High Resolution Melting analysis) [20] analysis are more sensitive and convenient than DS, however they are also time- and labor-consuming [21], and not readily available to most clinicians, often requiring that the tumor sample be sent to a reference laboratory, potentially resulting treatment delays.

We have developed a fully automated genetic analyzer AMDS which includes processes for DNA extraction/purification, DNA amplification (PCR), mutation detection by Invader® chemistry [22], [23], and genotype interpretation. AMDS can call a mutation status automatically in 70 minutes after addition of a sample (*e.g.,* extracted genomic DNA or tissue sample homogenate) to the cartridge. Here, we report a feasibility study of AMDS for detecting somatic *KRAS*, *BRAF* and *PI3KCA* mutations in CRC tissues by comparison with DS in a double-blind manner. We first evaluated the sensitivity of the AMDS using a titration assay with artificially constructed plasmid DNA. A clinical performance study was then conducted to further assess the accuracy, specificity and sensitivity of the system in comparison with DS. In addition, cloning-sequencing analysis was conducted in order to validate the discordant mutational status between AMDS and DS. The versatility of the system in detecting mutations from tissues with different fixatives (fresh frozen and FFPE) was also evaluated. In addition, we tested the capability of the system in a fully automated mode: from DNA extraction to mutation detection, using a minimal amount (>1 mg) of frozen CRC tissue.

## Materials and Methods

### Plasmid DNA

The targeted mutations were 7 nonsynonymous point mutations (G12A, G12C, G12D, G12R, G12S, G12V and G13D) in exon 2 of the *KRAS* gene, one synonymous point mutation (V600E) in exon 15 of the *BRAF* gene and 5 nonsynonymous point mutations in the exon 9 helical domain (E542K, E545K, E545G) and exon 20 kinase domain (H1047L, H1047R) of the *PIK3CA* gene, all common mutations in human CRC. These mutants and wild-types were PCR amplified and cloned into the plasmid pCR®2.1 (Invitrogen, CA, USA), and the synthesized mutant and wild-type templates were verified by sequencing. The length of all plasmid DNA including the 300 bp target sequence was 4.2 kb. The synthesized plasmid DNAs were suspended in TE buffer and stored at −20°C before use.

### CRC Specimen Section

CRC tissues of frozen specimen sections (n = 89) and FFPE specimen sections (n = 70) used in this study were from the Human Tissue Resource Center of the University of Chicago. All samples were diagnosed as colon or rectal cancer by hematoxylin and eosin stain. All tissues were primary CRC tissue surgically removed prior to other clinical treatments. Tissues were sliced to an approximate size of 1.0 cm^2^ × 10 µm by microtome. The sliced section samples used for this study were not performed by manual microdissection (MMD). No further information including demographic and clinical data were requested for these samples. The study has been reviewed and approved by the Institutional Review Board of the University of Chicago.

### AMDS

**Figure 1:**
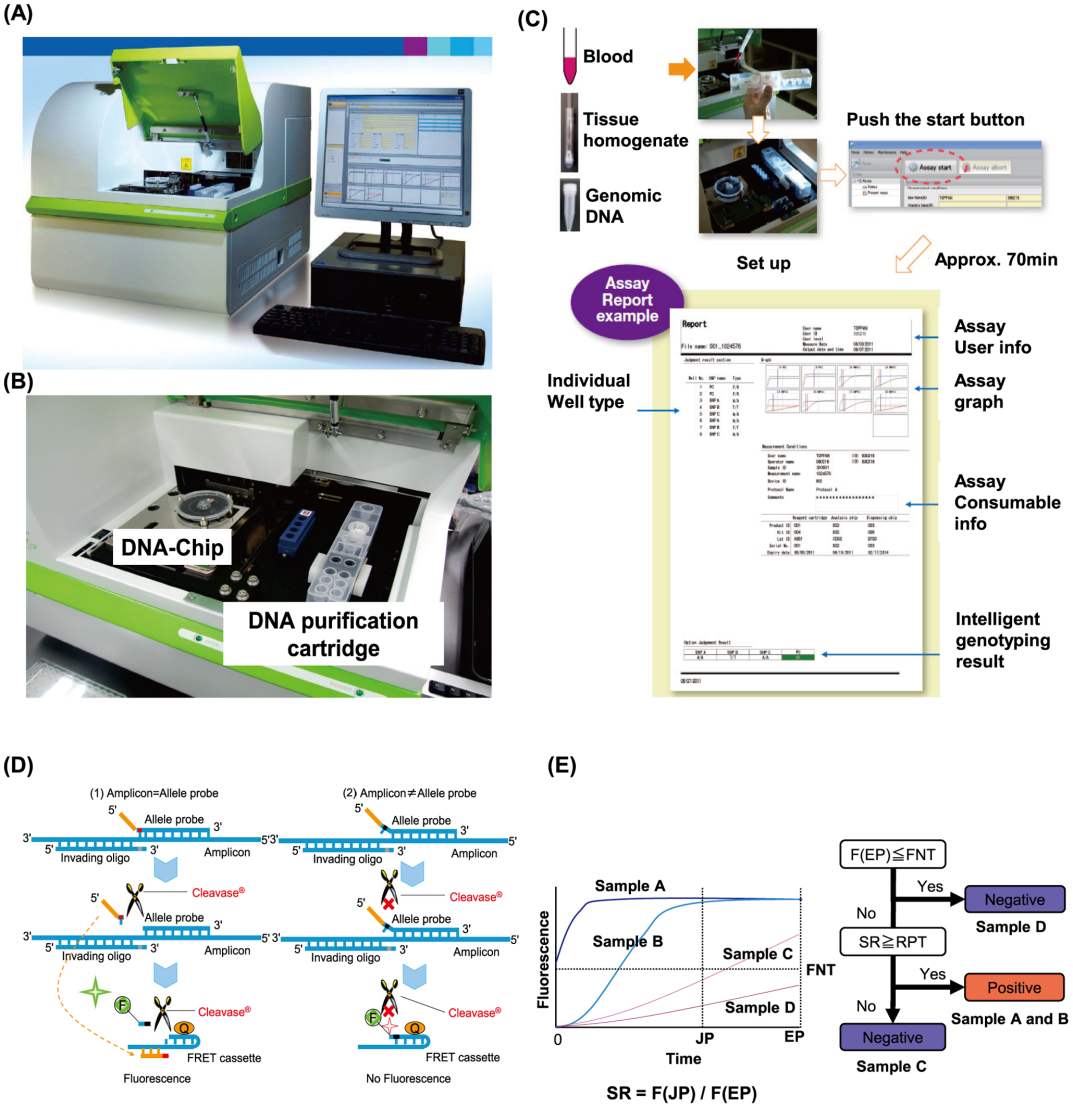
AMDS mutation detection system. (A), (B) AMDS system (DNA-chip, DNA purification cartridge and devise ) (C) Assay flow of AMDS. (D) Principle of Invader® chemistry (E) genotyping algorithm of AMDS. EP: End point time, JP: Judging point time, FNT: Fluorescence strength of Negative threshold, F(EP): Fluorescence strength at EP, F(JP): Fluorescence strength at JP, SR: Signal ratio, RPT: Positive ratio threshold.

AMDS is a fully automated genetic analytical system based on a DNA-chip which has 23 reaction wells containing reagents for PCR and Invader® assays (Figure 1A and 1B). When a user adds a sample (whole blood, purified DNA or tissue homogenate) to the DNA purification cartridge and starts the attached software, AMDS performs DNA extraction, transfers the DNA to the chip, performs PCR and the Invader® assay, reads the results, and displays judging result in about 70 minutes. Assay flow of mutation detection is shown in Figure 1C. In step 1, DNA is extracted and purified by the DNA purification cartridge; in step 2, the purified DNA fluid sample is transferred to the DNA-Chip; in step 3, InvaderPlus® (PCR and Invader® reaction continuously in the same tube) is conducted; in the last step, AMDS reports a genotyping result of the sample. InvaderPlus® was performed under the following conditions: denature for 2 min at 93°C, followed by 30 or 35 cycles of 31 seconds at 93°C and 16 seconds at 66°C, and Taq polymerase deactivation for 2 minutes at 97°C, followed by 10 minutes of signal detection at 61°C. Fluorescence signal of FAM (Fluorescence aminohexyl) was monitored in channel F1 at 520 nm with excitation of 490 nm, and fluorescence signal of RED (Redmond Red) was monitored in channel F2 at 595 nm with excitation of 580 nm.

### DNA-chip and DNA Purification Cartridge

All DNA chips and DNA purification cartridges were manufactured in a clean room at the level of ISO class 8. Required reagents mixture (1.99 µl) for a DNA chip containing 0.1 µl of 1 M MOPS buffer (pH 7.7) (DOJINDO LABORATORIES, Kumamoto, Japan), 0.05 µl of 10 mM each deoxyribonucleoside triphosphate (Roche, CA, USA), 0.96 µl of 1 M trehalose (Hayashibara, Okayama, Japan) aqueous solution, 0.60 µl of 20 × oligo mix, 0.22 µl of 5.0 U/µl Hawk Taq polymerase (Roche, CA, USA), 0.04 µl of 15,000 U/µl Cleavase (Hologic, WI, USA) were dispensed and dried in wells of a DNA chip. 20 × oligo mixture was prepared with 0.06 µl of 100 µM forward primer, 0.06 µl of 100 µM reverse primer, 0.06 µl of 50 µM of FAM-FRET (Fluorescence resonance energy transfer) cassette (Hologic, WI, USA), 0.06 µl of 50 µM of RED-FRET cassette (Hologic, WI, USA), 0.06 µl of D.W. (Distilled Water: Lonza, Basel-Stadt, Switzerland), and a set of 0.06 µl of 10 µM invading oligonucleotide plus 0.06 µl of 100 µM allele probe for both wild type and mutant type. Oligonucleotide sequences used in this study are shown in Table S1.

Nucleic acid was purified using a DNA purification cartridge containing a glass filter. DNA is adsorbed to silica in the presence of a chaotropic salt [24], [25]. After the purification process, 270 µl of DNA sample containing MgCl_2_ (6.25 mM) and NaCl (15 mM) was injected into the DNA-chip.

### The Principle of InvaderPlus® Assay for the Mutation Detection

InvaderPlus® is Invader® chemistry-based (Figure 1D) mutation detection assay, in which PCR and Invader reaction are carried out consecutively in single tube. After the PCR amplification step, DNA polymerase is heat inactivated, and Invader® reaction detects the mutation in the PCR product. Invading oligo nucleotide and allele probe bind to the PCR product forming invasive cleavage structure (1). If the sequence of the allele probe is fully matched with the PCR product, Cleavase® cuts the probe causing the release of arm. The released arm binds to the complementary sequence of the FRET cassette. Finally, Cleavase® cuts a FRET cassette, and separates fluorescence dye modified nucleotide (F) from the residual FRET cassette which contains quencher (Q) at the 3′ end. These reactions are cycled, and cause signal amplification. On the contrary, when the sequence of the probe has one-base mismatch with the PCR product at its 5′ end where the invading oligo nucleotide and allele probe have one base overlapping structure, Cleavase® cannot cut the allele probe. As a result, the consequent reaction does not take place, and the FRET cassette is intact (2). Two FRET cassettes are distinguished by use of different fluorescent dyes.

### Algorithm of Genotyping

The principle and flow chart of genotyping by AMDS is shown in Figure 1E. When Invader® assay has completed, the genotyping software compares the fluorescent signal strength at EP (End point time) described as F (EP), and FNT (fluorescent strength of negative threshold). If F (EP) is less than FNT, the sample is considered as negative (*e,g*, Sample D) for the mutation. If F (EP) is not less than FNT, then its signal ratio (SR), which is denoted by the following equation, is calculated.
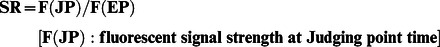



SR represents a reaction efficiency of Invader® assay. If SR of a sample is larger than RPT (Ratio Positive Threshold), the sample is considered as positive for the specific mutation (*e.g*. sample A and B), but if not, the sample is considered as negative (*e.g.* sample C). Each RPT for all mutations detected in this clinical study was defined with 5% mutant 95% wild-type mixture plasmid DNA (Table S2.).

### Direct Sequencing (DS)

For the DS, the following primers were used to amplify the *KRAS* gene: 5′-GAATGGTCCTGCACCAGTAA-3′ (F: Forward primer), 5′-GTGTGACATGTTCTAATATA GTCA-3′ (R: Reverse primer), *BRAF* gene: 5′-TGCTTGCTCTGATAGGAAAATG-3′ (F), 5′- AGCATCTCAGGGCCAAAAAT-3′ (R) and *PIK3CA* gene: 5′-ATGATGCTTGGCTCTGG AAT-3′ (F), 5′-GGTCTTTGCCTGCTGAGAGT-3′ (R). The length of each PCR product was *KRAS*: 214 bp, *BRAF*: 228 bp, *PIK3CA* ^ex9^: 269 bp and *PIK3CA* ^ex20^: 273 bp. PCR products were cycle-sequenced using the Big dye terminator v1.1/3.0 cycle sequencing kit (Life technologies, CA, USA) according to the manufacture’s instruction. Sequence reactions were then subjected to electrophoresis on an Applied Biosystems 3730×l DNA Analyzer (Life technologies, CA, USA).

### Titration Study using Plasmid DNA and Genomic DNA

**Figure 2:**
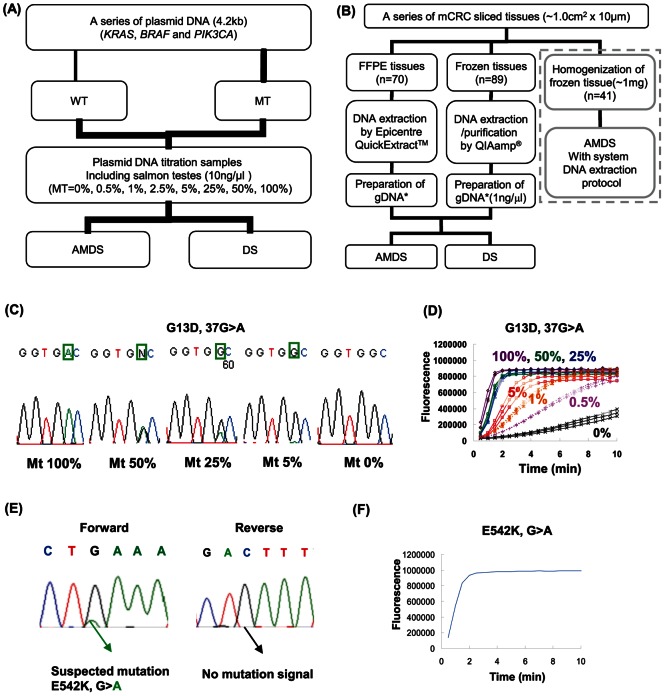
Evaluation of AMDS. (A) Plasmid DNA titration study. Plasmid DNA was constructed for total 13 different mutants and 6 wild-type (*KRAS*: 1, *BRAF*: 1, *PIK3CA*: 2). (B) Clinical performance study. 70 FFPE sliced tissues and 89 Frozen sliced tissues were tested. Genomic DNA was extracted from FFPE sliced tissue by Epicentre® QuickExtract™. Genomic DNA was purified from frozen sliced tissue by QIAamp® DNA Micro kit. Also, as surrounded by dotted lines, about 1 mg of frozen sliced tissue was homogenized and used for fully automated mutation analysis. (C) Titration study of *KRAS* G13D mutation detection by DS. The electropherograms were taken for different mutant-wild mixture of plasmid DNA (10 fg/reaction). (D) Titration study for *KRAS* G13D mutation detection by AMDS. The graph shows the merged InvaderPlus® reaction data (n = 3) for different mutant-wild mixture for *KRAS* G13D detection by AMDS (◊; mt 100%, ○; mt 50%, △; mt 25%, Υ; mt 5%, *; mt 1%, +; 0.5% and ×; mt 0%). Amount of plasmid DNA was 10 fg/well. (E) Electropherogram of forward and reverse analysis of same sample (ID = 56754). (F) InvaderPlus® reaction of a sample (ID = 56754) by AMDS.

To evaluate the mutation detection sensitivity of AMDS, a titration study of plasmid DNA was conducted. Titration samples (amount of plasmid DNA: 1 fg/well, 10 fg/well and 100 fg/well) were prepared as shown in Figure 2A. The sample mixtures contained 30 µl of plasmid DNA, 12 µl of 500 mM NaCl, 18 µl of 100 mM MgCl_2_, and 240 µl of D.W. Each plasmid DNA sample contains 100 ng/well of Salmon testes single stranded DNA (Sigma-Aldrich, MO, USA). Also, the contribution of back ground signal was checked with Novagen® human female genomic DNA (EMD biosiences, CA, USA) (1 ng/well, 10 ng/well and 100 ng/well).

### Clinical Performance Study

The experimental design for the clinical performance study of AMDS is shown in Figure 2B. Genomic DNA from frozen specimen sections (n = 89) was extracted and purified by the QIAmp® DNA Micro kit (Qiagen, CA, USA), and adjusted to 10 ng/µl. The sample loaded onto the DNA chip contained 30 µl of 10 ng/µl genomic DNA, 12 µl of 500 mM NaCl and 18 µl of 100 mM MgCl_2_, and 240 µl of D.W. Genomic DNA from FFPE sections (n = 70) was extracted using the QuickExtract™ FFPE DNA Extraction Kit [Epicentre® (an Illumina® company), WI, USA] according to the manufacturer’s instruction, and prepared as a × 50 dilution sample. Loading sample for the DNA chip contained 30 µl of extracted DNA (× 50 dilution), 12 µl of 500 mM NaCl, 18 µl of 100 mM MgCl_2_, 240 µl of D.W.

### Cloning Analysis

Cloning analysis was conducted for samples (n = 14) which had discordant mutational status between AMDS and DS. Insert lengths of PCR products were 214 bp (*KRAS*), 228 bp (*BRAF*), 269 bp (*PIK3CA* ^ex9^) and 273 bp (*PIK3CA* ^ex20^). The primer sequences are shown in Table S3. PCR products were prepared by using GoTaq® DNA polymerase and PrimeSTAR® GXL DNA polymerase. PCR products were digested by restriction enzyme, and the fragments were inserted to pUC118/HincII and pMD19/EcoRV. Inserted clones were recognized by blue-white screening. Plasmid DNA was amplified by illustra TempliPhi DNA Amplification Kit (GE healthcare, Buckinghamshire, England). DS was performed by Applied Biosystems 3730×l DNA Analyzer.

### Fully Automated Somatic Mutations Detection by AMDS

Approximately 1 mg of frozen specimen sections (n = 41) were homogenized for 20 seconds by glass homogenizer, and 200 µl of D.W. was added. The homogenate was transferred to the DNA purification cartridge of AMDS, and fully automated mutation detection process for *KRAS* and *PIK3CA* mutations were conducted. The frozen samples which harbored *BRAF* V600E mutation were not included in this study due to the limited amount of DNA.

### Statistical Analysis

Statistical analysis was carried out for statistical power and κ coefficient tests [26] which were used to compare the occurrence and degree of concordance in the detection of *KRAS*, *BRAF* and *PIK3CA* between DS and AMDS.

## Results

### Comparing Mutation Detection Sensitivity between AMDS and DS in the Plasmid DNA Titration Study

To evaluate the sensitivity of the AMDS, we used serially diluted plasmid DNAs containing different ratios of mutant and wild type of *KRAS*, *BRAF* and *PIK3CA* genes. When the sample contained less than 25% of mutant DNA, the mutation peak in the DS electropherogram was not able to be distinguished from the background (Figure 2C). In contrast, AMDS clearly detected *KRAS* G13D mutations at a 5% level (maximum 0.5%) as shown Figure 2D.

### Comparing Sensitivity of Mutation Detection between AMDS and DS in the Clinical Performance Study

The mutation detection performance was compared between AMDS and DS with two sets of samples; fresh frozen tissues (n = 89) and FFPE samples (n = 70) from patients (n = 153) with CRC. Paired frozen and FFPE samples were available for six patients, and the rest of the samples were from independent patients. The comparison was performed in a double-blinded manner. As shown in Table 1–3, all *KRAS*, *BRAF* and *PI3KCA* mutations detected by DS in either frozen (total number of mutation, n = 41, 46.0%) or FFPE (n = 27, 38.5%) samples were also successfully (100%) detected by AMDS. There were no samples which were determined as mutants in DS while detected as wild-type in AMDS. In the samples detected as wild-types by DS, however, AMDS was able to detect additional mutants in the frozen (n = 8, 9.0%) and FFPE (n = 6, 8.6%) samples. As shown in Table 4, mutations in both *KRAS* and *PIK3CA* were detected by AMDS in 6 patients (6/153, 3.9%). Notably, among these coexisting mutations, all *PI3KCA* mutations were specifically E545K, while *KRAS* mutations varied.

**Table 1:** 2×2 comparison of *KRAS* mutations frequency in frozen and FFPE tissue sections (n = 159).

*KRAS* mutation (κ = 0.91, *P* = 0.96)								
Frozen tissues					FFPE tissues			
AMDS	DS				AMDS	DS		
	MT	WT	TOTAL			MT	WT	TOTAL
MT	31	1	32		MT	21	3	24
WT	0	57	57		WT	0	45	45
TOTAL	31	58	89		TOTAL	21	48	69*

MT = Mutant-type.

WT = Wild type.

κ = κ coefficient.

*P* = statistical power.

* = 1 *KRAS* analysis in the FFPE tissues were failed in DS due to noisy sequencing data.

**Table 2:** 2×2 comparison of *BRAF* mutations frequency in frozen and FFPE tissue sections (n = 159).

*BRAF* mutation (κ = 0.67, *P* = 0.97)								
Frozen tissues					FFPE tissues			
AMDS	DS				AMDS	DS		
	MT	WT	TOTAL			MT	WT	TOTAL
MT	2	1	3		MT	1	0	1
WT	0	86	86		WT	0	62	62
TOTAL	2	87	89		TOTAL	1	62	63*

MT = Mutant-type.

WT = Wild type.

κ = κ coefficient.

*P* = statistical power.

* = 7 *BRAF* analysis in the FFPE tissues were failed in DS due to noisy sequencing data.

**Table 3:** 2×2 comparison of *PIK3CA* mutations frequency in frozen and FFPE tissue sections (n = 159).

*PIK3CA* mutation (κ = 0.7, *P* = 0.94)								
Frozen tissues					FFPE tissues			
AMDS	DS				AMDS	DS		
	MT	WT	TOTAL			MT	WT	TOTAL
MT	8	6	14		MT	5	3	24
WT	0	75	75		WT	0	61	45
TOTAL	8	81	89		TOTAL	5	64	69*

MT = Mutant-type.

WT = Wild type.

κ = κ coefficient.

*P* = statistical power.

* = 1 *PIK3CA* analysis in the FFPE tissues were failed in DS due to noisy sequencing data.

**Table 4:** Multiple mutations (Frozen and FFPE tissue sections).

Sample ID#	Status	*KRAS*	*BRAF*	*PIK3CA*
60682	Frozen	G12D	–	E545K
63439	Frozen	G12V*	–	E545K*
41949	Frozen	G13D	–	E545K
41950	Frozen	G13D	–	E542K*/E545K*
7053316	FFPE	G12D	–	E545K*
60681	FFPE	G12D	–	E545K

* = DS called as wild-type.

Results of DS and AMDS for a discordant example are shown in Figure 2E and 2F. This sample was plausibly determined as a mutant according to DS only with forward primer analysis. Due to the poor sequencing signal in reverse primer analysis, this sample was considered as a wild-type. In contrast, AMDS had a clear mutation signal. All other discordant results (8 in frozen tissue, 6 in FFPE tissues) had very similar patterns (data not shown).

### Validation of Discordant Data by Cloning and Sequencing

**Figure 3:**
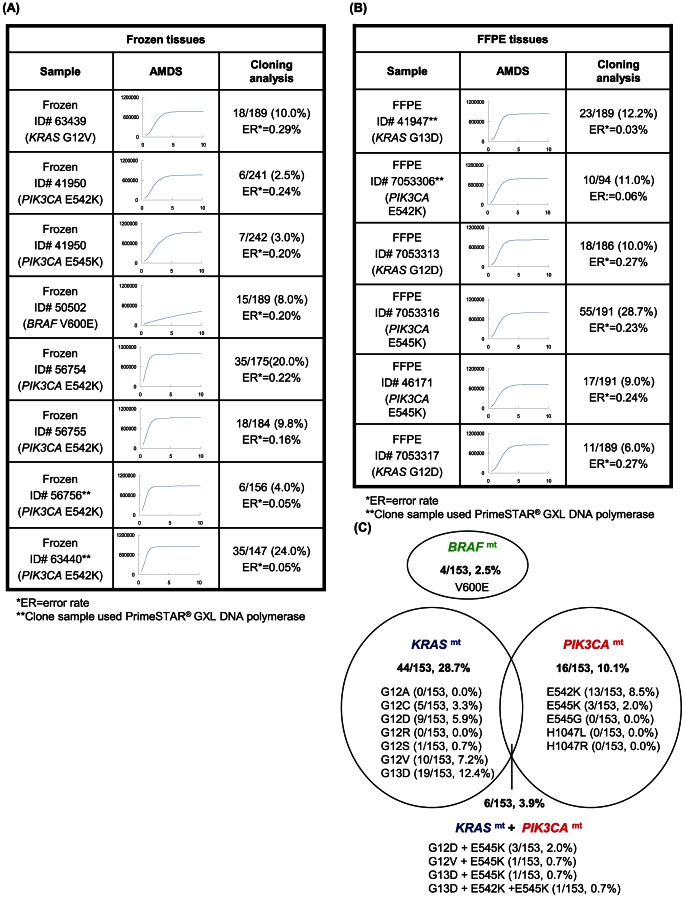
Cloning analysis and summary of genetic alteration in clinical samples. (A) Cloning analysis result of Frozen tissues. (B) Cloning analysis result of FFPE tissues.; PCR was performed for frozen ID = 56756, ID = 63440 and FFPE ID = 41947, ID = 7053306 samples with PrimeSTAR® GXL DNA polymerase. Other samples were performed PCR with GoTaq® DNA polymerase. Potential mutation frequency in a sample = (number of mutant sequence)/(number of successful sequence). If the frequency was higher than ER, the sample was considered as mutation positive. Note: It was not confirmed whether mutation rate of a sample was able to be quantified by the cloning analysis. (C) Venn diagram of *KRAS*, *BRAF* and *PIK3CA* mutations. In this graph, these frequencies of mutation are not calculated for samples but for patients. 6 samples were taken from same tissues and prepared for both frozen and FFPE slice. The AMDS analyses for these 6 samples showed same results for both Frozen and FFPE slice.

All samples which had discordant mutational status between AMDS and DS were validated by cloning analysis (Figure 3A, B). The accuracy of the cloning was also evaluated with error rate (ER) defined as the frequency of base alterations in the inserted region among the picked colonies as following equation; ER = the number of alterations/[(length of insert)×(number of successful clone sequence)]×100. The analyzed cloned regions did not have a hot spot for mutation other than the targeted points. Therefore the base alterations from consensus sequence were considered as misreadings by the DNA polymerase. ER of PrimeSTAR® GXL (0.03–0.06%), which has 3′→5′ exo-nuclease activity, was considerably lower than that of GoTaq® (0.16–0.29%). The frequency of all mutations of interest (Figure 3A, B) was much higher than ER (*p* = 1.04×10^−6^), indicating that the mutations in these samples were true mutations, and the discordance between the two methods was attributed to the lower sensitivity of DS. In this study, sample size was good enough since high degree of power in the *KRAS*, *BRAF* and *PIK3CA* mutation detection (*P* = 0.96, 0.97 and 0.94 respectively) by AMDS (Table 1–3). κ coefficient tests of *KRAS*, *BRAF* and *PIK3CA* (κ = 0.91, 0.67 and 0.70 respectively) mutations indicated low degree of coincidence between AMDS and DS.

Figure 3C summarizes the frequencies of mutations in all patients (n = 153) based on the AMDS detection. Mutation rates of *KRAS*, *BRAF* and *PIK3CA* were 28.7% (44/153), 2.5% (4/153) and 10.1% (16/153), respectively. The frequency of coexisting mutations in *KRAS* or *PIK3CA* was 3.9% (6/153).

**Figure 4:**
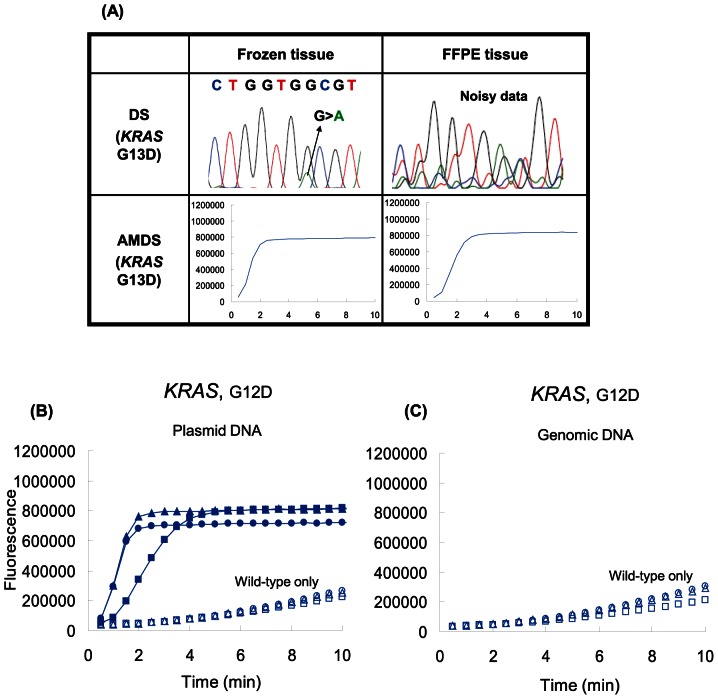
Assay versatility of AMDS. (A) DS and AMDS results for same tissue but different preservation. CRC tissues were prepared for both frozen and FFPE format. These samples were analyzed by both DS and AMDS. DS failed to call the genotype of the FFPE tissue due to noisy electrophenogram. The same sample was re-sequenced, and called as *KRAS* wild-type. (B) AMDS analysis results with different total amount of plasmid DNA. Solid symbols (•; 100 fg/well, ▪; 10 fg/well, ▴; 1fg/well ) indicate signal of samples containing 5% mutant, and empty symbols (○; 100 fg/well, □; 10 fg/well, △; 1 fg/well) indicate g of plasmid DNA roughly contain 210 copies. (C) AMDS analysis results with different amount of wild-type genomic DNA. (○; 100 ng/well, □; 10 ng/well, △; 1 ng/well).

AMDS and DS were compared for their robustness in mutation detection from DNA samples prepared using different methods. The Qiagen commercial DNA purification kit (QIAmp® DNA Micro kit) was used with frozen tissues, and another commercial DNA extraction kit (Quick Extract™) was used with FFPE tissues. Genotyping call rates for DS were 100.0% (89/89) and 74.3% (52/70) in frozen and FFPE samples, respectively, for the first attempt; whereas that of AMDS was 100.0% for both sample sets. Figure 4A shows a case of G13D mutation detection, in which the sample was taken from the same patient and processed in both frozen and FFPE slices. While the frozen sample has a clear electropherogram, the FFPE sample showed noisy signals. Eighteen samples were retested for DS, and 10 of these succeeded. However, 8 samples were not able to be analyzed in both the forward and reverse directions after multiple attempts. Out of these 8 samples, 7 samples could not be analyzed for the *BRAF* mutations, and 1 sample could not be analyzed for both *KRAS* and *PIK3CA* mutations. In contrast, AMDS perfectly called these samples as wild type for the *KRAS*, *BRAF* and *PIK3CA* genes.

The robustness of mutation detection for a wide range of sample concentrations was also tested for AMDS. Figure 4B shows the result of the plasmid DNA experiment. 5% of mutant DNA signal had clear separation from backgrounds for the range of 1 fg/well to 100 fg/well of total plasmid DNA, which corresponds to 10 copies to 1000 copies of target mutant DNA per reaction. Copy number of 1 fg/well plasmid DNA (210 copies) is equivalent to 0.63 ng/well of genomic DNA. The amount of genomic DNA (extracted frozen tissue) used in the clinical performance study was 10 ng/well, and that falls in the range of the above experiment. However, in order to check the background signal level, additional experiments with several amounts of human genomic DNA were conducted. Figure 4C indicates that the background signals are stable and almost identical to that of the plasmid DNA experiment (Figure 4B) for the range of 1 ng to 100 ng per well.

### Feasibility Study of a Fully Automated *KRAS*, *BRAF* and *PIK3CA* Mutation Detection by AMDS

In this experiment, crude extraction of frozen tissue samples (n = 41) was performed by manual crushing, and the raw homogenate was then used as a sample for fully automated mutation analysis by AMDS.

The results of the fully automated mutation analysis were perfectly concordant with the previous clinical performance studies (Table 5). In addition, AMDS was able to detect all mutants (3/41) that DS could not detect. Thus, AMDS could detect all *KRAS* (14/41, 34.1%) and *PIK3CA* (8/41, 19.5%) mutations even from these homogenate samples (∼1 mg tissue).

**Table 5:** Comparison between the Qiagen kit and AMDS DNA purification cartridge.

AMDS *v.s.* QIAGEN			
QIAmp® DNA Micro Kit	AMDS		
	WT	MT	TOTAL
WT	19	0	19
MT	0	22	22
TOTAL	19	22	41

WT = Wild-type.

MT = Mutant type.

## Discussion

This study evaluated AMDS in two series (Frozen and FFPE) of primary CRC tissues totaling 159 samples. Our data suggested AMDS has greater sensitivity and versatility than DS. The superior capability of our system may be attributable to the high sensitivity (>0.5%) and fidelity of the Invader® chemistry which contain signal amplification capability [22], [23]. This is of particular importance for mutation detection when mutant level is extremely low. The results shown in Figure 4B and 4C suggest AMDS can maintain a 5% mutation detection sensitivity with a very wide range (100 holds) of the sample concentration. Furthermore, Kotoura *et al*. previously reported that the PCR efficiency of DS and real-time PCR based assay on FFPE-DNA samples are suffered by DNA fragmentation [27]. As shown in Figure 3 and Figure 4A, results of mutation analysis via AMDS were supported by the cloning results, and the AMDS showed a significant superiority to DS in its sensitivity over the wide range of DNA amount and variety of fixation condition.

Additionally, the AMDS system succeeded in a fully automated detection of *KRAS*, *BRAF* and *PIK3CA* mutations with homogenized frozen CRC samples, which is advantageous for analyzing valuable samples such as biopsy tissues. The mutation detection procedure of AMDS is very simple and does not require experimental technique and experience.

One of the key things is that, in the cloning analysis, we observed that commonly used commercial Taq DNA polymerase has a not-so-low ER, while PrimeSTAR® GXL DNA polymerase with a 3′→5′ exo-nuclease activity (proofreading) has less ER. Therefore, when ultra high sensitivity is required for an assay such as mutation detection of cell-free DNA in blood samples (serum or plasma), high fidelity DNA polymerase should be used.

As result of evaluation for the clinical CRC tissues (n = 159) by using AMDS, mutation patterns detected in our samples are very consistent with what have previously reported [28]–[30]. We observed very interesting co-existence of multiple mutations among the three genes. For example, 6 out of 153 samples (3.9%) possessed double (one for triple) mutations, supporting the hypothesis of synergistic tumorigenesis between *KRAS* and *PIK3CA* [29], [31]. In addition, there were two samples which possessed both *KRAS* G13D and *PIK3CA* (E545K and/or E542K) mutations. The *KRAS* G13D mutation, unlike codon 12 mutations, may indicate a good response to cetuximab [9]. However, if *KRAS* G13D mutation coexists with the *PIK3CA* helical domain mutation, the benefit of anti-EGFR monoclonal antibody (moAb) treatment may be decreased due to activated PI3K pathway by RAS-PI3K interaction [13], [29]. Therefore, in order to optimize use of anti-EGFR moAb treatment for mCRC patients, not only *KRAS* but also *PIK3CA* mutation genotyping might be required. Furthermore, one sample (ID# 51950) had triple mutations including simultaneous “hot spot” mutations (E542K and E545K), and the cloning analysis confirmed that they are not on the same allele. Thus, it was speculated that each mutation occurred in the same cell on a different allele, or, the mutated alleles came from different cancer cells. Such an observation has not been reported in the Catalogue of Somatic Mutations in Cancer [1]. In addition, although the G13D mutation was detected by both DS and AMDS, neither E542K nor E545K mutations were detected by DS alone. Given that DS has similar sequencing sensitivity for all regions in our study, we deduce that the number of cells that possess the E542K and E545K mutation might be much less than that of G13D. Hence, our data suggests cancer heterogeneity in tumor tissues.

### Conclusions

AMDS has superior sensitivity and accuracy over DS, and is much easier to execute than conventional labor intensive manual mutation analysis. AMDS has great potential for POCT equipment for mutation analysis.

## Supporting Information

Table S1 Oligo DNA sequence used in this study.
(PPT)

Table S2 RPT used in clinical performance study.
(PPT)

Table S3 Primer sequence used for the cloning analysis.
(PPT)
