# Supplementary material for: A Novel Fully Automated Molecular Diagnostic System (AMDS) for Colorectal Cancer Mutation Detection
Source: PLoS One. 2013 May 9;8(5):e62989. doi: 10.1371/journal.pone.0062989 (PMC3650034; doi:10.1371/journal.pone.0062989)
Supplement: Table S1 — Oligo DNA sequence used in this study. (PPT) [file pone.0062989.s001.ppt]

## Slide 1
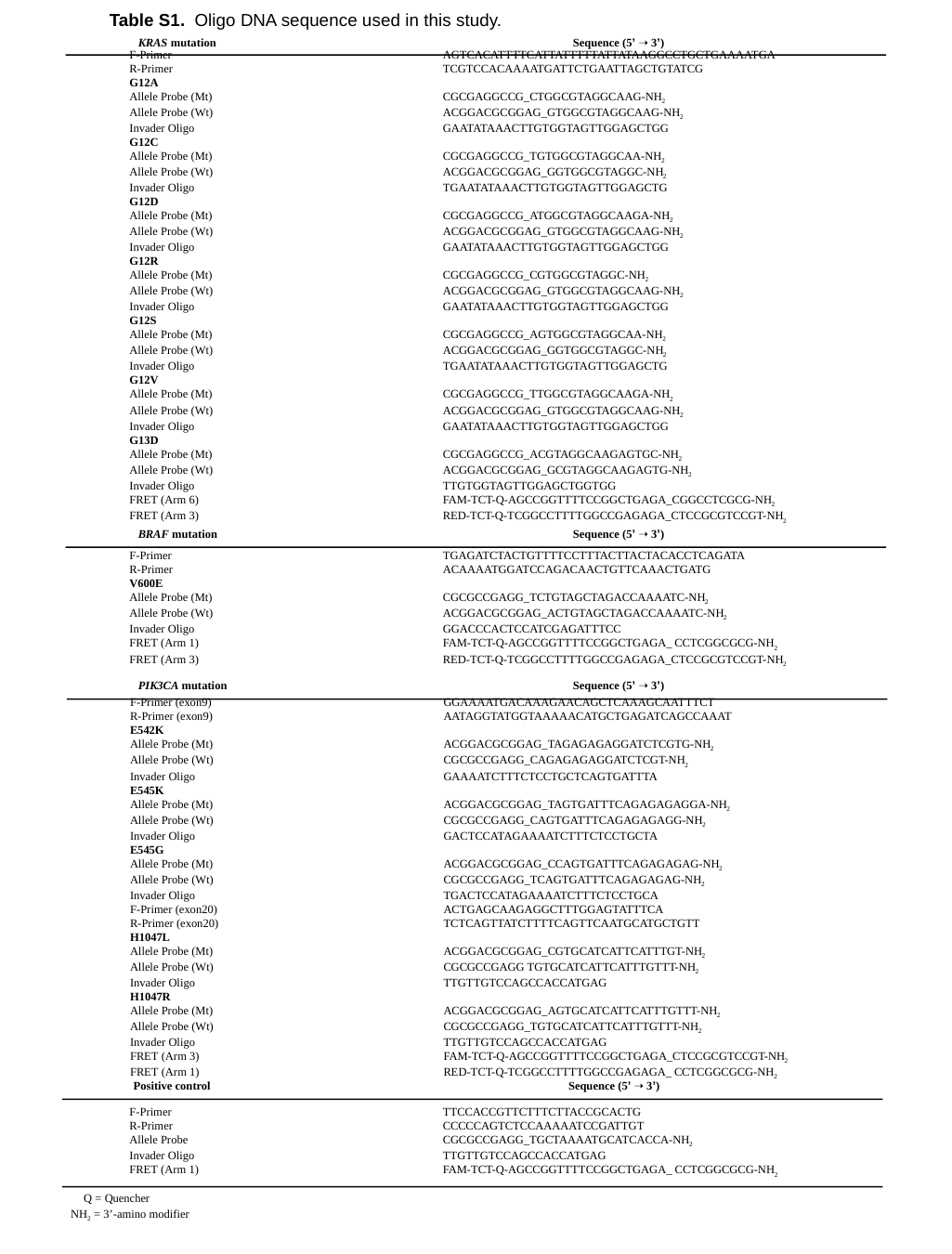

Table S1. Oligo DNA sequence used in this study.
KRAS mutation
Sequence (5’ ➝ 3’)
F-Primer	AGTCACATTTTCATTATTTTTATTATAAGGCCTGCTGAAAATGA
R-Primer	TCGTCCACAAAATGATTCTGAATTAGCTGTATCG
G12A
Allele Probe (Mt)	CGCGAGGCCG_CTGGCGTAGGCAAG-NH2
Allele Probe (Wt)	ACGGACGCGGAG_GTGGCGTAGGCAAG-NH2
Invader Oligo	GAATATAAACTTGTGGTAGTTGGAGCTGG
G12C
Allele Probe (Mt)	CGCGAGGCCG_TGTGGCGTAGGCAA-NH2
Allele Probe (Wt)	ACGGACGCGGAG_GGTGGCGTAGGC-NH2
Invader Oligo	TGAATATAAACTTGTGGTAGTTGGAGCTG
G12D
Allele Probe (Mt)	CGCGAGGCCG_ATGGCGTAGGCAAGA-NH2
Allele Probe (Wt)	ACGGACGCGGAG_GTGGCGTAGGCAAG-NH2
Invader Oligo	GAATATAAACTTGTGGTAGTTGGAGCTGG
G12R
Allele Probe (Mt)	CGCGAGGCCG_CGTGGCGTAGGC-NH2
Allele Probe (Wt)	ACGGACGCGGAG_GTGGCGTAGGCAAG-NH2
Invader Oligo	GAATATAAACTTGTGGTAGTTGGAGCTGG
G12S
Allele Probe (Mt)	CGCGAGGCCG_AGTGGCGTAGGCAA-NH2
Allele Probe (Wt)	ACGGACGCGGAG_GGTGGCGTAGGC-NH2
Invader Oligo	TGAATATAAACTTGTGGTAGTTGGAGCTG
G12V
Allele Probe (Mt)	CGCGAGGCCG_TTGGCGTAGGCAAGA-NH2
Allele Probe (Wt)	ACGGACGCGGAG_GTGGCGTAGGCAAG-NH2
Invader Oligo	GAATATAAACTTGTGGTAGTTGGAGCTGG
G13D
Allele Probe (Mt)	CGCGAGGCCG_ACGTAGGCAAGAGTGC-NH2
Allele Probe (Wt)	ACGGACGCGGAG_GCGTAGGCAAGAGTG-NH2
Invader Oligo	TTGTGGTAGTTGGAGCTGGTGG
FRET (Arm 6)	FAM-TCT-Q-AGCCGGTTTTCCGGCTGAGA_CGGCCTCGCG-NH2
FRET (Arm 3)	RED-TCT-Q-TCGGCCTTTTGGCCGAGAGA_CTCCGCGTCCGT-NH2
BRAF mutation
Sequence (5’ ➝ 3’)
F-Primer	TGAGATCTACTGTTTTCCTTTACTTACTACACCTCAGATA
R-Primer	ACAAAATGGATCCAGACAACTGTTCAAACTGATG
V600E
Allele Probe (Mt)	CGCGCCGAGG_TCTGTAGCTAGACCAAAATC-NH2
Allele Probe (Wt)	ACGGACGCGGAG_ACTGTAGCTAGACCAAAATC-NH2
Invader Oligo	GGACCCACTCCATCGAGATTTCC
FRET (Arm 1)	FAM-TCT-Q-AGCCGGTTTTCCGGCTGAGA_ CCTCGGCGCG-NH2
FRET (Arm 3)	RED-TCT-Q-TCGGCCTTTTGGCCGAGAGA_CTCCGCGTCCGT-NH2
PIK3CA mutation
Sequence (5’ ➝ 3’)
F-Primer (exon9)	GGAAAATGACAAAGAACAGCTCAAAGCAATTTCT
R-Primer (exon9)	AATAGGTATGGTAAAAACATGCTGAGATCAGCCAAAT
E542K
Allele Probe (Mt)	ACGGACGCGGAG_TAGAGAGAGGATCTCGTG-NH2
Allele Probe (Wt)	CGCGCCGAGG_CAGAGAGAGGATCTCGT-NH2
Invader Oligo	GAAAATCTTTCTCCTGCTCAGTGATTTA
E545K
Allele Probe (Mt)	ACGGACGCGGAG_TAGTGATTTCAGAGAGAGGA-NH2
Allele Probe (Wt)	CGCGCCGAGG_CAGTGATTTCAGAGAGAGG-NH2
Invader Oligo	GACTCCATAGAAAATCTTTCTCCTGCTA
E545G
Allele Probe (Mt)	ACGGACGCGGAG_CCAGTGATTTCAGAGAGAG-NH2
Allele Probe (Wt)	CGCGCCGAGG_TCAGTGATTTCAGAGAGAG-NH2
Invader Oligo	TGACTCCATAGAAAATCTTTCTCCTGCA
F-Primer (exon20)	ACTGAGCAAGAGGCTTTGGAGTATTTCA
R-Primer (exon20)	TCTCAGTTATCTTTTCAGTTCAATGCATGCTGTT
H1047L
Allele Probe (Mt)	ACGGACGCGGAG_CGTGCATCATTCATTTGT-NH2
Allele Probe (Wt)	CGCGCCGAGG TGTGCATCATTCATTTGTTT-NH2
Invader Oligo	TTGTTGTCCAGCCACCATGAG
H1047R
Allele Probe (Mt)	ACGGACGCGGAG_AGTGCATCATTCATTTGTTT-NH2
Allele Probe (Wt)	CGCGCCGAGG_TGTGCATCATTCATTTGTTT-NH2
Invader Oligo	TTGTTGTCCAGCCACCATGAG
FRET (Arm 3)	FAM-TCT-Q-AGCCGGTTTTCCGGCTGAGA_CTCCGCGTCCGT-NH2
FRET (Arm 1)	RED-TCT-Q-TCGGCCTTTTGGCCGAGAGA_ CCTCGGCGCG-NH2
Positive control
Sequence (5’ ➝ 3’)
F-Primer	TTCCACCGTTCTTTCTTACCGCACTG
R-Primer	CCCCCAGTCTCCAAAAATCCGATTGT
Allele Probe	CGCGCCGAGG_TGCTAAAATGCATCACCA-NH2
Invader Oligo	TTGTTGTCCAGCCACCATGAG
FRET (Arm 1)	FAM-TCT-Q-AGCCGGTTTTCCGGCTGAGA_ CCTCGGCGCG-NH2
Q = Quencher
NH2 = 3’-amino modifier
