# Supplementary material for: A Novel Fully Automated Molecular Diagnostic System (AMDS) for Colorectal Cancer Mutation Detection
Source: PLoS One. 2013 May 9;8(5):e62989. doi: 10.1371/journal.pone.0062989 (PMC3650034; doi:10.1371/journal.pone.0062989)
Supplement: Table S2 — RPT used in clinical performance study. (PPT) [file pone.0062989.s002.ppt]

## Slide 1
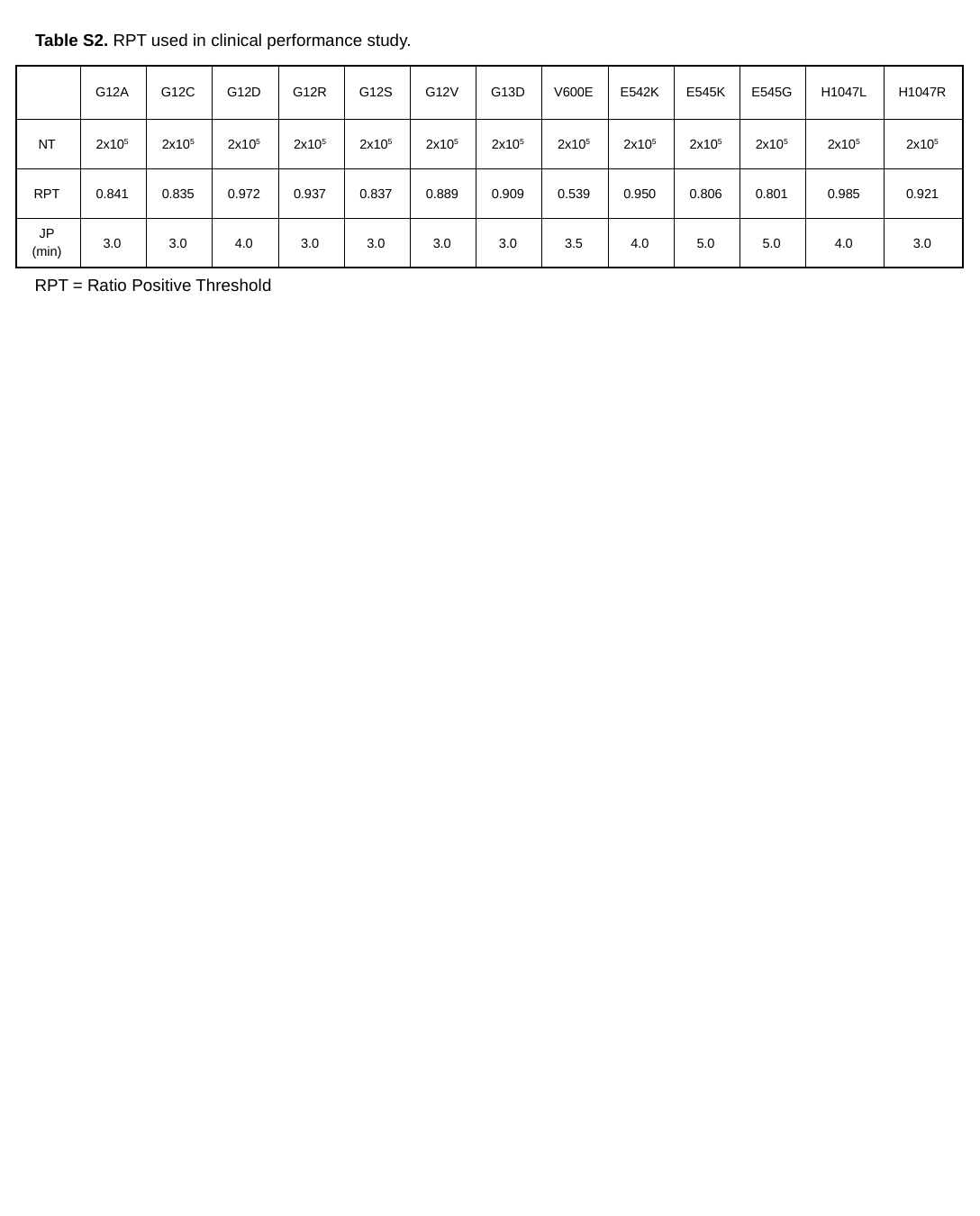

Table S2. RPT used in clinical performance study.
| | G12A | G12C | G12D | G12R | G12S | G12V | G13D | V600E | E542K | E545K | E545G | H1047L | H1047R |
| --- | --- | --- | --- | --- | --- | --- | --- | --- | --- | --- | --- | --- | --- |
| NT | 2x10^5^ | 2x10^5^ | 2x10^5^ | 2x10^5^ | 2x10^5^ | 2x10^5^ | 2x10^5^ | 2x10^5^ | 2x10^5^ | 2x10^5^ | 2x10^5^ | 2x10^5^ | 2x10^5^ |
| RPT | 0.841 | 0.835 | 0.972 | 0.937 | 0.837 | 0.889 | 0.909 | 0.539 | 0.950 | 0.806 | 0.801 | 0.985 | 0.921 |
| JP (min) | 3.0 | 3.0 | 4.0 | 3.0 | 3.0 | 3.0 | 3.0 | 3.5 | 4.0 | 5.0 | 5.0 | 4.0 | 3.0 |
RPT = Ratio Positive Threshold
