# Supplementary material for: A Novel Fully Automated Molecular Diagnostic System (AMDS) for Colorectal Cancer Mutation Detection
Source: PLoS One. 2013 May 9;8(5):e62989. doi: 10.1371/journal.pone.0062989 (PMC3650034; doi:10.1371/journal.pone.0062989)
Supplement: Table S3 — Primer sequence used for the cloning analysis. (PPT) [file pone.0062989.s003.ppt]

## Slide 1
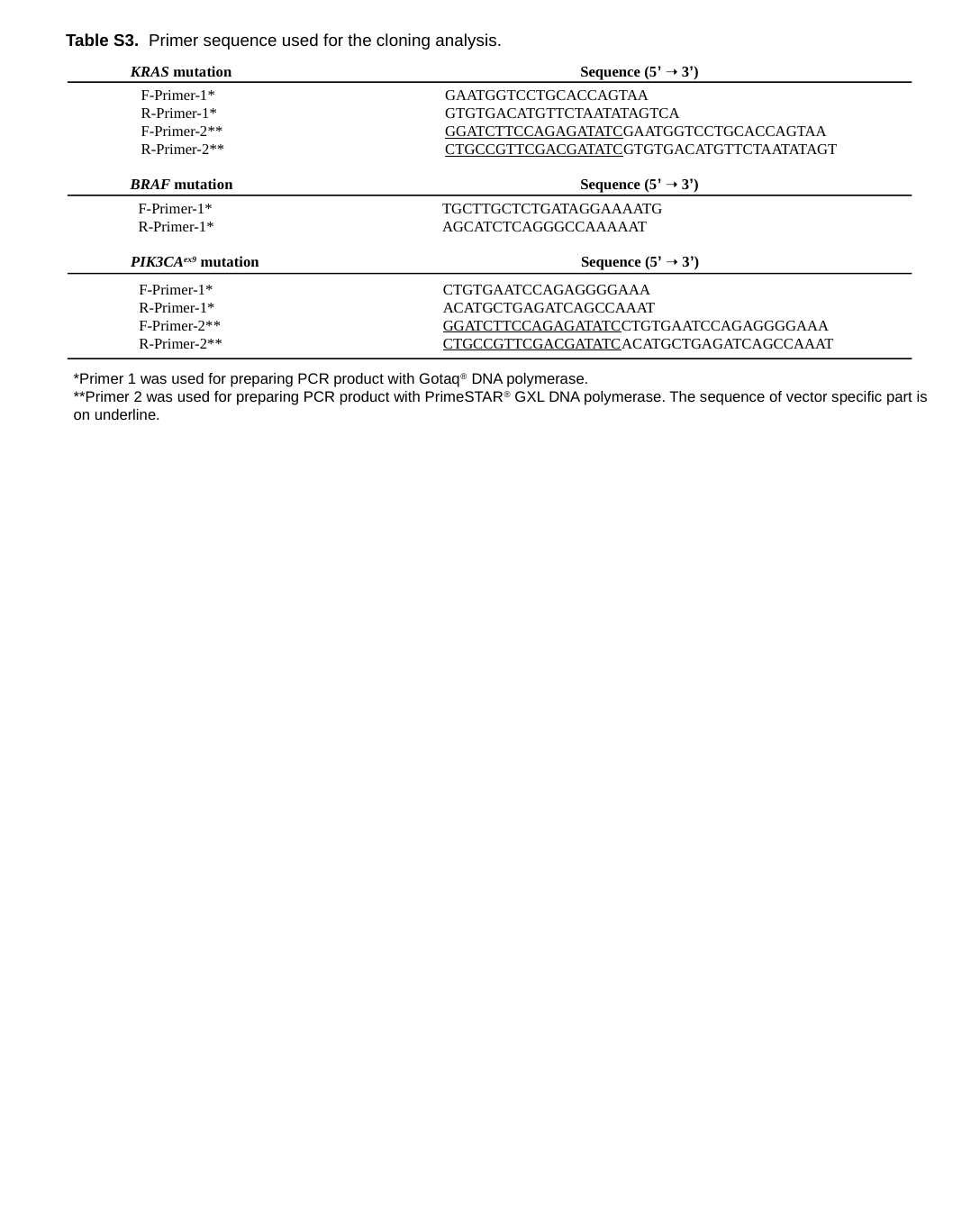

Table S3. Primer sequence used for the cloning analysis.
KRAS mutation
Sequence (5’ ➝ 3’)
F-Primer-1*	GAATGGTCCTGCACCAGTAA
R-Primer-1*	GTGTGACATGTTCTAATATAGTCA
F-Primer-2**	GGATCTTCCAGAGATATCGAATGGTCCTGCACCAGTAA
R-Primer-2**	CTGCCGTTCGACGATATCGTGTGACATGTTCTAATATAGT
BRAF mutation
Sequence (5’ ➝ 3’)
F-Primer-1*	TGCTTGCTCTGATAGGAAAATG
R-Primer-1*	AGCATCTCAGGGCCAAAAAT
PIK3CAex9 mutation
Sequence (5’ ➝ 3’)
F-Primer-1*	CTGTGAATCCAGAGGGGAAA
R-Primer-1*	ACATGCTGAGATCAGCCAAAT
F-Primer-2**	GGATCTTCCAGAGATATCCTGTGAATCCAGAGGGGAAA
R-Primer-2**	CTGCCGTTCGACGATATCACATGCTGAGATCAGCCAAAT
*Primer 1 was used for preparing PCR product with Gotaq® DNA polymerase.
**Primer 2 was used for preparing PCR product with PrimeSTAR® GXL DNA polymerase. The sequence of vector specific part is
on underline.
